# Increased Hemoglobin Oxygen Affinity With 5-Hydroxymethylfurfural Supports Cardiac Function During Severe Hypoxia

**DOI:** 10.3389/fphys.2019.01350

**Published:** 2019-10-30

**Authors:** Alfredo Lucas, Eilleen S. Y. Ao-ieong, Alexander T. Williams, Vivek P. Jani, Cynthia R. Muller, Ozlem Yalcin, Pedro Cabrales

**Affiliations:** ^1^Department of Bioengineering, University of California, San Diego, San Diego, CA, United States; ^2^School of Medicine, Koç University, Istanbul, Turkey

**Keywords:** 5-HMF, hemoglobin, allosteric effectors, oxygen delivery, conductance catheter, cardiac function, left ventricle

## Abstract

Acclimatization to hypoxia or high altitude involves physiological adaptation processes, to influence oxygen (O_2_) transport and utilization. Several natural products, including aromatic aldehydes and isothiocyanates stabilize the R-state of hemoglobin (Hb), increasing Hb-O_2_ affinity and Hb-O_2_ saturation. These products are a counter intuitive therapeutic strategy to increase O_2_ delivery during hypoxia. 5-Hydroxymethylfurfural (5-HMF) is well known Amadori compound formed during the Maillard reaction (the non-enzymatic browning and caramelization of carbohydrate-containing foods after thermal treatment), with well documented effects in Hb-O_2_ affinity. This study explores the therapeutic potential of 5-HMF on left ventricular (LV) cardiac function (LVCF) during hypoxia. Anesthetized Golden Syrian hamsters received 5-HMF i.v., at 100 mg/kg and were subjected to stepwise increased hypoxia (15, 10, and 5%) every 30 min. LVCF was assessed using a closed chest method with a miniaturized conductance catheter via continuous LV pressure-volume (PV) measurements. Heart hypoxic areas were studied using pimonidazole staining. 5-HMF improved cardiac indices, including stroke volume (SV), cardiac output (CO), ejection fraction (EF), and stroke work (SW) compared to the vehicle group. At 5% O_2_, SV, CO, EF, and SW were increased by 53, 42, 33, and 51% with 5-HMF relative to vehicle. Heart chronotropic activity was not statistically changed, suggesting that differences in LV-CF during hypoxia by 5-HMF were driven by volume dependent effects. Analysis of coronary blood flow and cardiac muscle metabolism suggest no direct pharmacological effects from 5-HMF, therefore these results can be attributed to 5-HMF-dependent increase in Hb-O_2_ affinity. These studies establish that naturally occurring aromatic aldehydes, such as 5-HMF, produce modification of hemoglobin oxygen affinity with promising therapeutic potential to increase O_2_ delivery during hypoxic hypoxia.

## Introduction

Food products are subjected to thermal treatments to assure microbiological safety, eliminate enzymatic activities, and to obtain desirable sensory properties. 5-Hydroxymethylfurfural (5-HMF) is naturally formed during the thermal treatment of carbohydrate-containing foods because of the Maillard reaction (non-enzymatic browning or caramelization). 5-HMF is known as an Amadori compound, resulting from tautomerization of the N-glycoside of an aldose or the glycosylamine to the corresponding 1-amino-1-deoxy-ketose via an immine intermediate (Hodge, 1955; Kurti and Czako, 2005). Therefore, 5-HMF generally is known as an indicator of heating for a wide range of foods (Rada-Mendoza et al., 2002). Several factors influence the formation of 5-HMF in foods, including carbohydrate content, pH, temperature of treatment, and water content (Hodge, 1955). In general, monosaccharides (i.e., fructose or glucose) are substrates for 5-HMF production (Rada-Mendoza et al., 2002). The toxicological relevance of 5-HMF at very high concentrations is minimal (Ulbricht et al., 1984). However, recent studies showed that 5-HMF orally, intraperitoneally, or intravenously has a high bioavailability (Abdulmalik et al., 2005). 5-HMF has a high red cell membrane permeability, where it increases hemoglobin (Hb) O_2_ affinity by stabilizing the R-state of Hb, which has higher O_2_ affinity (Abdulmalik et al., 2005). 5-HMF binds covalently with Hb to form a high-affinity imine Hb adduct in a symmetrical fashion with the NH_2_-terminal α Valine-1 of Hb, allosterically shifting the Hb-O_2_ equilibrium curve at relatively low 5-HMF concentrations (Abdulmalik et al., 2005).

Acute mountain sickness (AMS) is frequently diagnosed in unacclimated individuals who ascend to 2300 m of altitude or higher, and studies have shown that an upward of 25% of travelers experience symptoms (Roach and Hackett, 2001; Basnyat and Murdoch, 2003). The severity of symptoms can vary with the degree of the altitude of acclimatization and are a result of the many physiological responses to a hypoxic environment. Exposure to these hypoxic conditions reduces O_2_ transport from the air to the tissues by blood. The fate of the cardiac tissue is ultimately determined the balance between myocardial O_2_ delivery (MDO_2_) and consumption (MVO_2_). MDO_2_ is defined by coronary blood flow and O_2_ saturation, whereas MVO_2_ is determined by myocardial O_2_ tension, heart rate (HR), contractile state, basal resting metabolism, and the work performed by the heart (Sonnenblick et al., 1968). Therefore, during hypoxic hypoxia, coronary blood flow needs to increase to preserve MVO_2_. The normal myocardium is known to have good tolerance to hypoxic conditions. When subjects were exposed to a simulated altitude of 4500 m by adjusting the fraction of inspired O_2_ (FiO_2_), they experienced an increase in coronary blood flow to maintain cardiac tissue oxygenation (Wyss et al., 2003). However, subjects with a history of cardiac diseases showed the opposite reaction; at a simulated altitude of 2500 m, these patients were observed to have an 18% reduction in coronary blood flow, whereas their healthy counterparts showed a 10% increase in coronary blood flow (Wyss et al., 2003). Thus, acclimatization to hypoxia requires increases in coronary blood flow to preserve O_2_ delivery to the cardiac tissue. Inability to increase cardiac output (CO) during hypoxic hypoxia would limit O_2_ delivery to the brain and other vital organs.

Oxygen transport and delivery is carried out by red blood cells (RBCs), where O_2_ reversibly binds to Hb. When FiO_2_ is reduced (hypoxic hypoxia), arterial pO_2_ and Hb-O_2_ saturation decrease, limiting tissue O_2_ delivery (MairbÄurl et al., 1986). Exposure to moderate hypoxia leads to a right shift in the Hb-O_2_ binding curve, facilitating O_2_ offload to tissues typically mediated by an increase in the RBCs 2,3-diphosphoglycerate concentration (2,3-DPG), decrease in the pH of blood (Bohr Effect), and an increase in CO_2_ (Haldane Effect) (Hebbel et al., 1978). Decreased Hb-O_2_ affinity during hypoxic hypoxia leads to a more drastic reduction in O_2_ uptake in the lungs, decreasing arterial O_2_ saturation, and limiting the adaptive response to hypoxia. However, birds and mammals that live at high altitudes have adapted to hypoxia by genetically increasing Hb affinity for O_2._ Their increased O_2_ affinity Hb favors O_2_ uptake in the lungs, thus increasing arterial O_2_ saturation during hypoxic hypoxia (Black and Tenney, 1980; Storz, 2007).

Left shifting the Hb-O_2_ equilibrium curve (OEC) secures O_2_ at the pulmonary level during hypoxic hypoxia, increasing O_2_ carrying capacity (OCC), and preserving O_2_ delivery independent of blood flow. This study aims to evaluate the effect of increased Hb-O_2_ affinity with 5-HMF in left ventricle (LV) cardiac function (LVCF) during hypoxia. We hypothesize that increasing Hb-O_2_ affinity with exogenously administered 5-HMF would increase arterial O_2_, allowing for superior coronary O_2_ delivery and preservation of LVCF at extreme hypoxia, and support systemic O_2_ delivery. Previous studies by our group determined that animals treated with 5-HMF had a decrease in blood P50 (partial pressure of O_2_ at which Hb is 50% saturated with O_2_) by up to 15 mmHg, providing increased microvascular O_2_ delivery during anemia and 5% O_2_ hypoxia (Yalcin and Cabrales, 2012). In this study, we aim to further inquire into the mechanical response of the heart, using a miniaturized conductance catheter to measure the ventricular PV changes during hypoxic challenges. Changes in oxygen distribution in the microcirculation due to 5-HMF were studied using hyperspectral images (HSI) of hamster window chamber models. Finally, potential effects of 5-HMF in cardiac muscle metabolism were assessed through isolated mouse heart studies.

## Materials and Methods

Animal handling and care followed the NIH Guide for Care and Use of Laboratory Animals. The experimental protocol was approved by the UCSD Institutional Animal Care and Use Committee.

### Animal Preparation

**FIGURE 1:**
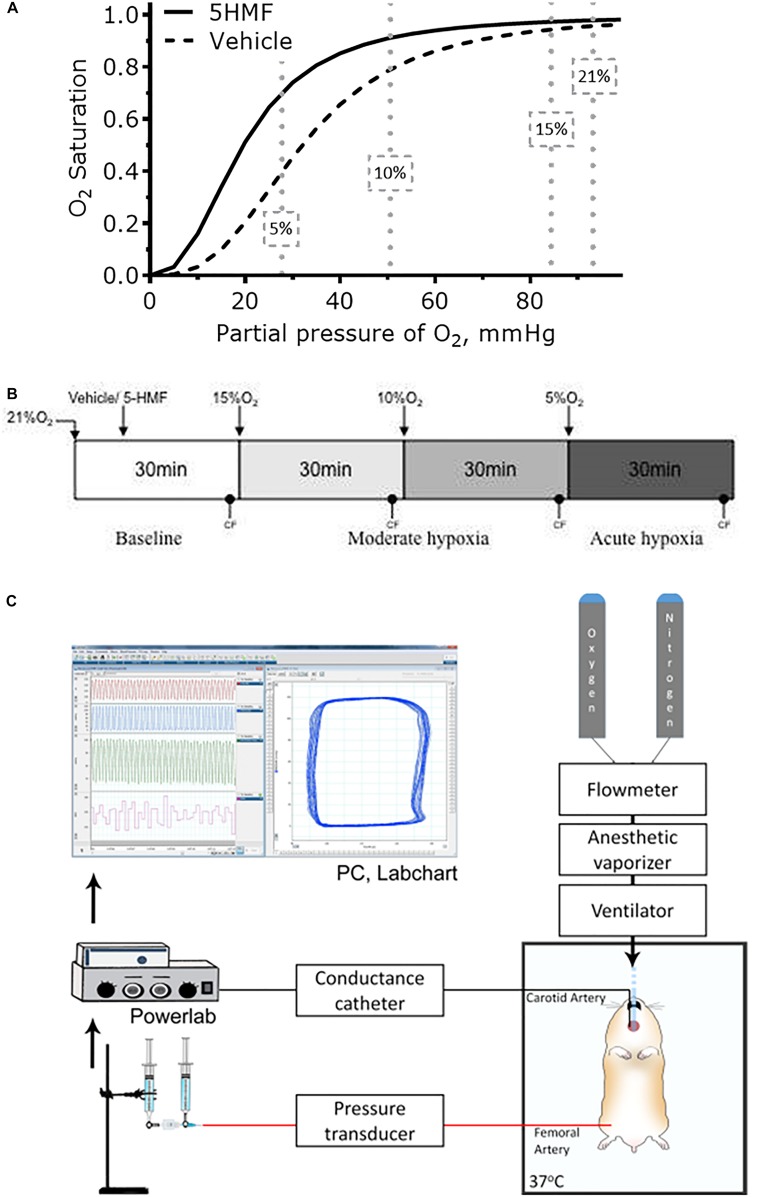
**(A)** Changes in blood Hb-O_2_ equilibrium during normoxia and hypoxia. 5-HMF increased blood Hb-O_2_ equilibrium in a dose dependent matter. At 10 and 5% O_2_, arterial Hb-O_2_ saturation should be higher with a left shift in the Hb-O_2_ dissociation curve (Kurti and Czako, 2005). **(B)** Hypoxia scheme followed in the experiments. After 5-HMF or the vehicle, animals were exposed to hypoxia by decreasing the fraction of inspired O_2_ (FiO_2_)stepwise from 15% to 10%, and then to 5% O_2_. Each hypoxic step was maintained for 30 min. **(C)** Representation of the experimental setup. Hamster was maintained on a 37°C heated platform. Tracheotomy was performed to achieve consistent mechanical ventilation and volatile anesthesia administration. Compressed gases (100% O_2_ and 100% N_2_) were connected to a flowmeter tonometer to adjust achieve desired FiO_2_. Flowmeter tonometer provided gases to anesthesia vaporizer and ventilator. Conductance catheter (MPVS3000, Millar Instruments) was inserted through the carotid artery and advanced to the LV. Femoral artery was catheterized for blood samples and blood pressure measurements. Jugular vein was catheterized for fluid infusions. CF and MAP measurements were collected using data acquisition hardware (PowerLab 8/30, AD Instruments), attached to a personal computer, and stored for off-line analysis using PVAN software (PVAN 3.6, Millar Instruments). Parallel volume was calibrated at the end of the experiment via IV injection of 10 μL hypertonic saline (10% NaCl) (Pacher et al., 2008).

Studies were performed in male Golden Syrian hamsters (80–90 g, Charles River Laboratories, Boston, MA, United States). The experimental protocol is similar to that reported in a previous study by our group (Ao-Ieong et al., 2017). Animals were initially anesthetized using sodium pentobarbital (40 mg/kg IP). Subsequently, the animals were implanted with left jugular vein and left femoral artery catheters. A LV conductance catheter was introduced through the right carotid artery. A tracheotomy was performed (polyethylene-90 tube), and animals were ventilated (TOPO ventilator, Kent Scientific, Torrington, CT, United States) at a respiration rate of 90 breaths/min and peak inspiratory pressure of 20 cmH_2_O to aid with breathing in the supine position. Animals were placed on a heating pad to maintain core body temperature at 37 °C and after instrumentation, volatile anesthesia (0.6%/vol Isoflurane, Drägerwerk AG, Lübeck, Germany) was administered using a vaporizer connected to the ventilator to preserve the depth of anesthesia during the experimental protocol. Depth of anesthesia was continually verified via toe pinch, and, if needed, isoflurane concentration was increased by 0.1%/vol to prevent animal discomfort. Experimental setup is presented in Figure 1C.

### Inclusion Criteria

Animals were suitable for the experiments if: (i) mean arterial blood pressure (MAP) was above 80 mmHg at baseline, (ii) stroke volume (SV) was above 18 μL at baseline, (iii) systemic hematocrit was above 45%, (iv) HR was above 350 bpm at baseline, and (v) CO was above 8 mL/min at baseline.

### Cardiac Function

A 1.4F pressure volume (PV) conductance catheter (SPR-839, Millar Instruments, TX, United States) was inserted into the LV using the closed chested method (Pacher et al., 2008). Briefly, the PV catheter inserted through the exposed right carotid artery and slowly advanced, passing through the aortic valve, into the LV. Pressure and volume signals were acquired continuously (MPVS3000, Millar Instruments, Houston, TX, United States and PowerLab 8/30, AD Instruments, Colorado Springs, CO, United States). Left ventricular volume was measured in conductance units (relative volume unit, RVU) and converted to absolute blood volume (μL) at the end of the experiment (Pacher et al., 2008; Doyle et al., 2016). Parallel volume was calibrated at the end of the experiment via IV injection of 10 μL hypertonic saline (10% NaCl) (Pacher et al., 2008).

### Cardiac Pressure-Volume Indices

Cardiac function was analyzed using PVAN software (PVAN 3.6, Millar Instruments, TX, United States). Cardiac function parameters were calculated from 20 to 25 cardiac cycles at each time point. SV, stroke work (SW), CO, ejection fraction (EF), cardiac contractility (dP/dt/VEd), and arterial elastane (Ea) were directly calculated in the PVAN software. Systemic vascular resistance (SVR) was calculated as: $\text{SVR}=\frac{\text{MAP}}{\text{CO}}$. Internal energy utilization (IEU) was used as a measure of internal metabolism of the LV (Doyle et al., 2013), and it was calculated as $\text{IEU=}\left({\left[{\text{V}}_{\text{es}}{\text{-V}}_{\text{0}}\right]}^{*}{\text{P}}_{\text{es}}\right)/2$, where V_es_ is the end systolic volume, V_0_ is the ESPV volume axis intercept, and P_es_ is the end systolic pressure. Using the assumption that end systolic PV curve intercept is small, V_0_ was set to zero for the calculations. LV mechanical efficiency (VME) was defined and calculated as: $\text{VME}=\frac{\text{SW}}{\text{PVA}}$,where PVA is the total PV area given by: PVA = (IEU + *SW*). Oxygen delivery (DO_2_) was calculated as: DO_2_ = CO^∗^(1.34^∗^HCT^∗^SaO_2_ + 0.003^∗^pO_2_).

### Hypoxia Protocol

After surgery, animals were kept under normoxia for 30 min, after which baseline measurements were taken. Then, twelve animals (*N* = 12) were randomly assigned to receive either 100 uL of sterile saline with 100 mg/kg 5-HMF (Sigma-Aldrich, St. Louis, MO, United States) or only saline 20 min before exposure to hypoxia. This dosage is based in previous *in vivo* mice studies, where a single 100 mg/kg oral dose was efficacious in prolonging survival under severe hypoxia (Abdulmalik et al., 2005). The same study found that a maximum plasma concentration of 5-HMF at 30 min after infusion for doses of 100 mg/kg, therefore by initiating hypoxia at 20 min post infusion ensures that the maximum plasma concentration is achieved during hypoxia. Six animals were assigned to each group. Animals were subjected to 30 min challenge of 15% hypoxia, then to 30 min challenge of 10% hypoxia, and lastly to 30 min challenge of 5% O_2_ hypoxia. Cardiac function measurements were taken 25 min into respective hypoxic stage. Representation of experimental timeline is presented in Figure 1B.

### Systemic Hemodynamics Parameters

Mean arterial blood pressure and heart rate were recorded continuously from the femoral artery (PowerLab 8/30, AD Instruments, Colorado Springs, CO, United States). Hematocrit was measured from centrifuged arterial blood samples taken in heparinized capillary tubes. Hb content was determined spectrophotometrically (B-Hemoglobin; Hemocue, Stockholm, Sweden). Arterial blood was collected in heparinized glass capillary tubes (50 uL) and immediately analyzed for arterial oxygen tension (pO_2_), arterial carbon dioxide partial pressure (pCO_2_), and pH (RapidLab 248; Bayer, Norwood, MA, United States). Ventricular PV measurements were continuously measured (PowerLab 8/30, AD Instruments, Colorado Springs, CO, United States).

### Oxygen Equilibrium Curve (OEC)

Blood OECs were measured using a TCS Hemox Analyzer (TCS Scientific Company) at the end of the experiment. To measure changes in the O_2_ affinity, blood samples were diluted 100X-fold in TCS Hemox buffer. In the Hemox Analyzer, samples were first saturated with compressed air (21% O_2_) and then deoxygenated with pure nitrogen. The absorbance of Hb at 570 nm and at 560 nm were recorded as a function of the O_2_ tension. The P50 were calculated using regression analysis with the TCS Hemox analyzer software.

### Histology

Immunohistochemistry staining for pimonidazole bound to hypoxic zones in vital tissues during hypoxia was completed via IV injection of the hypoxic marker Hypoxyprobe-1 (40 mg/kg pimonidazole, Hypoxyprobe, Burlington, MA, United States) and 5 mg/kg Hoechst 33342 (Invitrogen, Carlsbad, CA, United States) diluted in PBS (total volume 100 μL) 5 min before 10% hypoxia. The hearts were immediately excised after euthanasia and fixed with a 10% glutaraldehyde solution. Consecutive six parasternal short axis (PSS, cross-sectional “slice”) sections of the heart were created. Six to 10 random areas per slide were analyzed for positive pimonidazole staining.

### Oxygen Distribution During Acute Hypoxia

Golden Syrian hamsters (80–90 g, Charles River Laboratories, Boston, MA, United States) were implanted with dorsal skinfold window chamber following the same approach described in previous studies (Tsai et al., 2003; Yalcin and Cabrales, 2012). Prior to imaging, the animal was placed inside a restraining tube with the window chamber protruding out of the tube. A stage securing the tube was fixed to the microscope stage for intravital microscopy evalaution (BX51WI; Olympus, New Hyde Park, NY, United States). Arterioles and venules were mapped across the visible region of the chamber (Tsai et al., 2003), and their location was noted for future analysis with the hyperspectral system. After chamber mapping, the animal dorsal window was imaged using a Pika L hyperspectral imaging system (Resonon Inc., Bozeman, MT, United States) from which HSI of the microcirculation where acquired. The white reference reflectance spectra (*R*_ref_) of the system was calibrated using a white Teflon slab. The animal was allowed to rest in the tube environment for 20 min at normoxia before imaging. Images were taken at normoxia, and immediately after exposure to 10% oxygen for 1 min. Images were taken following the previously described approach at baseline and after 5-HMF (400 mg/kg). 5-HMF was given immediately after imaging the previous hypoxic trial, and 15 min were allowed before the next hypoxic trial.

Each HSI contained wavelength bands from 450 nm to 600 nm with a spacing of 5 nm between adjacent bands. Vessels were segmented from the background using a Gabor filter bank ranging from 0 to 360 degrees in the resulting image of the ratio between band 575 and band 485, which previous experiments have shown has maximum Hb contrast, independent of saturation. The saturation of Hb in the vessel was determined through a pixel-by-pixel least-squares spectral fit according to Equation 1, following an approach adapted from Hendargo et al. (2015).

(1) ε(λ)=b0+b1ε(λ)H⁢b+b2ε(λ)H⁢b⁢O2

Where *ε*(λ) is the absorbance spectra of any given pixel, given by $\varepsilon \,\left(\lambda \right)=\log\left(\frac{R_{\text{ref}}\,\left(\lambda \right)}{R\,\left(\lambda \right)}\right)$, where *R*_ref_ is the white reference reflectance spectra, and *R* is the reflectance spectra from the HSI. *ε*_*Hb*_ corresponds to the standard absorbance of deoxyhemoglobin and *ε*_*HbO*_2__ to the standard absorbance of oxyhemoglobin. The *b*_0_^–^*b*_2_ are the coefficients determined by least-squares, where *b*_0_ accounts for any offset, and *b*_1_ and *b*_2_ are proportional to the amount of deoxy- and oxyhemoglobin, respectively. Once the coefficients were determined, the saturation was calculated according to Equation 2:

(2) $$S\,O_{2}=\frac{b_{2}}{b_{1}+b_{2}}\times 100\%$$

### Heart Isolation and Perfusion

Studies were performed in ten male Sprague-Dawley rats (Harlan Laboratories, Indianapolis, IN, United States) weighing 200–250 g, with 5 rats were assigned to each group. Animal handling and care followed the NIH Guide for Care and Use of Laboratory Animals. The experimental protocol was approved by the UCSD Institutional Animal Care and Use Committee. Rats were heparinized (100 U i.p.) 15 min before anesthesia with 60 mg/kg of pentobarbital sodium (IP). Hearts were excised and placed in ice-cold Krebs-Henseleit bicarbonate (KHB) solution. All extraneous tissues were removed, and the aorta was cannulated with plastic cannula. After cannulation, the heart underwent a retrograde Langendorff perfusion (60 mmHg perfusion pressure) with KHB solution for, 15 min. At this time, the left atrium was connected to the preload reservoir by cannulating the pulmonary vein. The perfusate reservoir was used to control preload line, and was wrapped with a water jacket and heated to 38.7°C and, which resulted in a myocardial temperature around 37°C. Heart was switched from Langendorff to working mode and perfused at constant preload pressure of 15 mmHg LV worked against a hydrostatic column set at a height equivalent to a pressure of 50 mmHg. Pressure development in the aortic (afterload) line was measured using a 2.5-Fr transducer (Millar Instruments, Houston, TX, United States). Pressure measurements were recorded continually. HR was determined from the pressure traces. Coronary flow measurements were obtained by collecting the effluent dripping off the heart.

### Perfusate Solutions

The KHB solution used for the initial Langendorff perfusion consisted of 118.5 mM NaCl, 25 mM NaHCO3, 4.7 mM KCl, 1.2 mM MgSO4, 1.2 mM KH2PO4, 2.5 mM CaCl2, 0.5 mM EDTA, and 11 mM glucose and was gassed with 95% O2-5% CO2 (pH 7.4). The working heart solution used in this experiment was a modified KHB solution containing palmitate (0.5 mM) bound to 3% bovine serum albumin (BSA) as a metabolic substrate in addition to 11 mM glucose. Working heart solution was continuously gassed with various oxygen concentrations balanced with 5% CO_2_ to achieve set oxygen tensions in the solution. Hearts were studied with working heart solutions or working heart solution with 3 mM 5-HMF. Glucose and palmitate were used because they are the most abundant metabolic substrates for cardiac energy metabolism. They allow for the evaluation of glucose utilization (glycolysis and oxidation) and fatty acid oxidation in isolated working hearts perfused with isotopic glucose and palmitate.

### Measurement of Substrate Metabolism

Metabolism of isotopic labeled glucose was measured as outlined before (Saddik and Lopaschuk, 1991). All determinations of substrate metabolism were made in duplicate. Rates of glycolysis and glucose oxidation were measured simultaneously. Steady-state metabolic rates were calculated as the mean values when perfusate samples were collected from the working heart. Metabolic rates obtained for the various metabolic pathways were normalized for heart mass (dry wt). Glycolytic flux was determined by measuring the amount of 3H_2_O released from the metabolism of 5-[3H]glucose by the triosephosphate isomerase and enolase steps of the glycolytic pathway. To separate 3H_2_O from 5-[3H]glucose and [U-14C]glucose, perfusate samples were separated using an anion exchange columns. Briefly, a 100 μL sample was loaded on the column and eluted into scintillation vials with 0.8 mL of H_2_O. Samples were counted for 3H and 14C. Glucose oxidation was determined by measuring 14CO_2_ released by the metabolism of [U-14C] glucose. The 14CO_2_ released during glucose oxidation was trapped by bubbling the outflow air through 1M hyamine hydroxide. The 14CO_2_ left in the form of bicarbonate anion solution in the perfusion medium was released with KHB solution. Total 14CO_2_ production included the 14CO_2_ in the outflow air and the perfusion solution.

### Statistical Analysis

Results are presented as mean ± standard deviation. The measured hemodynamic, blood gases, pH and lactate parameters are presented as absolute values. The measured cardiac function parameters are presented relative to the baseline values. A ratio of 1.0 signifies no change from the baseline, whereas lower or higher ratios are indicative of changes proportionally lower or higher compared to baseline. The Grubbs’ method was used to assess closeness for all measured parameters at baseline. Statistically significant changes between groups and different FiO_2_ values were analyzed using two-way ANOVA with multiple comparisons. All statistics were calculated using GraphPad Prism 6 (GraphPad, San Diego, CA). Results were considered statistically significant if *P* < 0.05.

## Results

Twelve animals were included in the hypoxic challenge study, 5-HMF (100 mg/kg; *n* = 6); and Vehicle (*n* = 6). All animals tolerated the experimental protocol without signs of stress or discomfort. Animals passed the Grubbs’ test, ensuring that all parameters at baseline were within a similar population (*P* > 0.3). After administration of 5-HMF or the vehicle, animals were exposed to hypoxia by decreasing the % O_2_ stepwise from 15% to 10%, and then to 5% O_2_. Each hypoxic step was maintained for 30 min (Figure 1B).

### Changes in Blood O_2_ Affinity

Administration of 5-HMF or exposure to hypoxia caused no significant changes in the Hct and Hb (Table 1). Single administration of 5-HMF at 100 mg/kg decreased the P50 from 32.2 ± 0.5 mmHg to 21.6 ± 1.2 mmHg in the 5-HMF group (*P* < 0.05), but did not have any effect in the vehicle groups [P50 = 32.4 ± 0.7 mmHg (Table 1), representative shift in P50 is shown in Figure 1A]. Single administration of 5-HMF at 400 mg/kg, as used to determine arteriolar and venular Hb-O_2_ saturation in the dorsal window using HSI, decreased the P50 to 17.2 ± 0.9 mmHg. Changes in P50 with 5-HMF were maintained throughout the entire duration of the protocol.

**TABLE 1:** Blood O_2_ Carrying capacity and O_2_ affinity.

	**Baseline**	**Groups**	
Hct (%)	49.1 ± 1.1	5-HMF	48.9 ± 0.7
		Vehicle	49.3 ± 0.9
Hb (g/dl)	14.8 ± 0.8	5-HMF	14.7 ± 0.6
		Vehicle	14.9 ± 0.5
P50 (mmHg)	32.2 ± 0.5	5-HMF	21.6 ± 1.2∗
		Vehicle	32.4 ± 0.7

*Values represent means ± SD. Baseline included all the animals in the study. No significant differences were detected at baseline between groups, before treatment. ^∗^represents statistically significant differences (*P* < 0.05) compared to vehicle according to an unpaired *t*-test.*

### Systemic and Blood Chemistry

**FIGURE 2:**
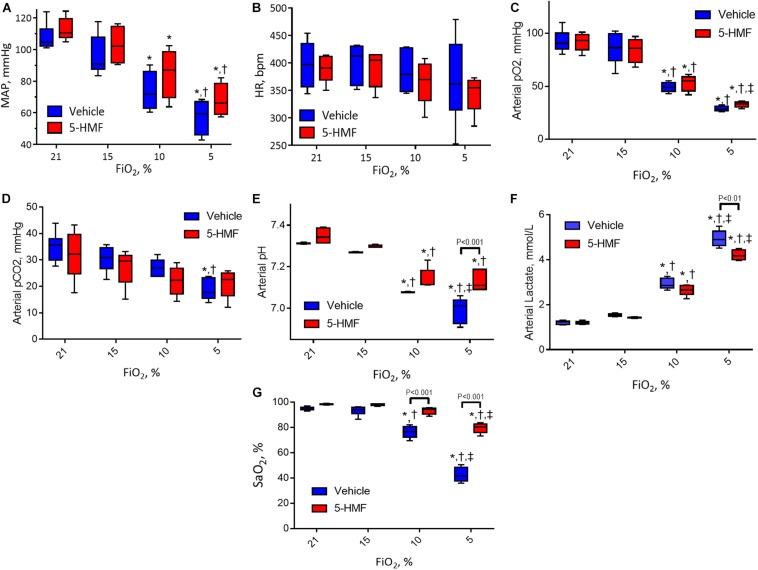
**(A)** Mean arterial blood pressure (MAP), **(B)** heart rate (HR), **(C)** arterial oxygen tension (pO_2_), **(D)** arterial partial pressure of carbon dioxide (pCO_2_), **(E)** arterial pH, and **(F)** arterial lactate during the long-term hypoxia protocol. **(G)** Shows the HbO_2_ saturation calculated from the pO_2_ and the Hill equation for HbO_2_ dissociation with and without 5-HMF. The p50 value used for the 5-HMF treated and vehicle groups are shown in Table 1. Symbols represent statistically significant differences (*P* < 0.05): ^∗^ relative to 21% FiO_2_, † relative to 15% FiO_2_, and ‡ relative to 10% FiO_2_.

Systemic hemodynamics and blood gases are presented in Figure 2. MAP remained unchanged when first exposed to moderate hypoxia of 15% O_2_ and decreased as hypoxia became more severe in both groups (Figure 2A). At 10% and 5% O_2_, the animals treated with 5-HMF showed higher MAP than the vehicle group, but no statistically significant differences were seen. Similarly, no significant changes in HR were observed between hypoxia levels or treatment groups, however, the 5-HMF group had a lower median HR at 5% O_2_ (Figure 2B). Both pO_2_ and pCO_2_ decreased with hypoxia (Figures 2C,D). 5-HMF resulted in slightly higher pCO_2_ and pO_2_ compared to vehicle, however, no statistical difference was observed between the groups. As expected, pH decreased with decreased O_2_ concentration, and the vehicle group had a significantly more acidic pH at 10 and 5% FiO_2_ concentrations relative to the 5-HMF group at the same O_2_ concentrations (Figure 2E). Blood lactate levels increased with hypoxia in both groups, and the 5-HMF group had significantly lower lactate compared to the vehicle group at 5% O_2_ (*P* < 0.05, Figure 2F). Arterial O_2_ saturation (SaO_2_) decreased in both groups; however, the vehicle group had a statistically significant lower SaO_2_ at 10 and 5% O_2_ compared to the 5-HMF group (*P* < 0.05). At 15 and 10% FiO_2_ there were no significant differences in SaO_2_ for the 5-HMF group, whereas the vehicle group had statistically significant lower SaO_2_ at 10%.

### Cardiac Function

**FIGURE 3:**
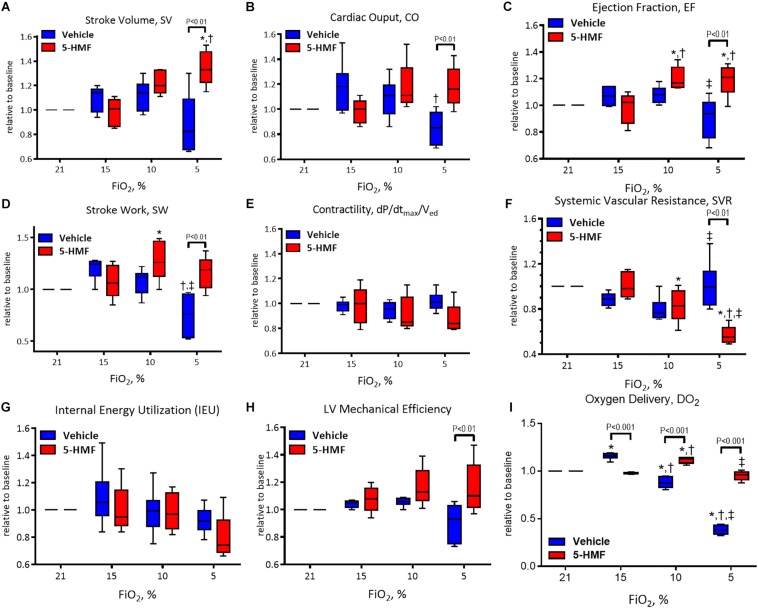
Left ventricle (LV) cardiac function (CF) measurements during hypoxia. **(A)** Stroke volume (SV), **(B)** cardiac output (CO), **(C)** ejection fraction (EF), **(D)** stroke work (SW), **(E)** contractility (dP/dt_max_/V_ed_), **(F)** systemic vascular resistance (SVR), **(G)** internal energy utilization (IEU), **(H)** left ventricular mechanical efficiency, and **(I)** oxygen delivery (DO_2_). Symbols represent statistically significant differences (*P* < 0.05): ^∗^ relative to 21% FiO_2_, † relative to 15% FiO_2_, and ‡ relative to 10% FiO_2_.

All LV indices of function are presented normalized to normoxia and are summarized in Figure 3.

Stroke volume is presented in Figure 3A and remained unchanged during 15 and 10% O_2_ hypoxia; however, a statistically significant decrease in SV was observed in vehicle group compared to the 5-HMF group at 5% O_2_ hypoxia (*P* < 0.05). The 5-HMF group increased SV by 38% from normoxia during 5% O_2_ hypoxia (*P* < 0.05), while the vehicle group decreased SV 19% for from normoxia at the same hypoxic level.

Cardiac output is presented in Figure 3B. During mild hypoxia (15% O_2_), the vehicle group showed an 18% increase in CO from normoxia. However, as hypoxia become more severe, CO decreased with the severity of hypoxia. At 5% O_2_, the CO of the vehicle group was 79% of that in the normoxia. On the other hand, the CO of the 5-HMF group was preserved during hypoxia, and increased by 11 and 19% from normoxia at 10 and 5% O_2_ hypoxia, respectively. CO was statistically higher for the 5-HMF group compared to the vehicle group at 5% O_2_ hypoxia (*P* < 0.05). EF is presented in Figure 3C. EF was statistically higher for the 5-HMF group compared to the vehicle group at 5% O_2_ hypoxia (*P* < 0.05). SW is presented in Figure 3D and was preserved as hypoxia increased in the 5-HMF groups, whereas in the vehicle group, SW decreased with the severity of the hypoxia. SW was statistically higher for the 5-HMF group compared to the vehicle group at 5% O_2_ hypoxia (*P* < 0.01). Contractility, in terms of dP/dtmax normalized by V_ed_ is presented in Figure 3E, and remained unchanged during the experiment in both groups. SVR is presented in Figure 3F, and was observed to decrease with the severity of hypoxia in the 5-HMF groups, but no difference in SVR was observed in the vehicle group compared to normoxia. SVR was statistically lower for the 5-HMF group compared to the vehicle group at 5% O_2_ hypoxia (*P* < 0.01). IEU is presented in Figure 3G, and it remained unchanged during the experiment in both groups. LV mechanical efficiency is presented in Figure 3H and remained unchanged during 15 and 10% O_2_ hypoxia; however, LV mechanical efficiency was significantly lower for the vehicle group compared to the 5-HMF group at 5% O_2_ (*P* < 0.01). DO_2_ is shown in Figure 3I. At 15%, the vehicle group had a statistically significant increase in DO_2_ relative to normoxia, and when compared to the 5-HMF group at 15% FiO_2_. At 10% FiO_2_, the 5-HMF group had a statistically significant increase in DO_2_ relative to normoxia for the same group, and relative to the vehicle group at 15% hypoxia, which had a statistically significant decrease in DO_2_. Finally, at 5% FiO_2_, the DO_2_ for the 5-HMF group decreased back to baseline levels when compared to the 10% FiO_2_ case. When compared to the 5-HMF group at 5% and the vehicle group at normoxia, 15 and 10%, the vehicle group at 5% FiO_2_ had the lowest DO_2_. The normoxia values for the different cardiovascular variables for the vehicle and 5-HMF groups are shown in Table 2. The majority of the parameters shown in Figure 3 are derived from those variables.

**TABLE 2:** Cardiovascular Parameters at Normoxia (21% FiO_2_).

	**Vehicle (*n* = 6)**	**5-HMF (*n* = 5)**
HR (BPM)^∗^	397.0 ± 41.6	490.6 ± 25.4
MAP (mmHg)	107.6 ± 8.4	112.6 ± 7.2
Ves (μL)	16.0 ± 0.6	16.6 ± 0.9
Ved (μL)^∗^	36.1 ± 2.1	36.2 ± 2.2
SV (μL)	20.1 ± 2.4	19.6 ± 2.3
Pes (mmHg)	123.3 ± 8.6	120.8 ± 4.7
Ped (mmHg)	7.2 ± 0.4	7.2 ± 0.5

*Values represent means ± SD. ^∗^ represents statistically significant differences (*P* < 0.05) between the two groups according to an unpaired *t*-test.*

### Pimonidazole Staining

**FIGURE 4:**
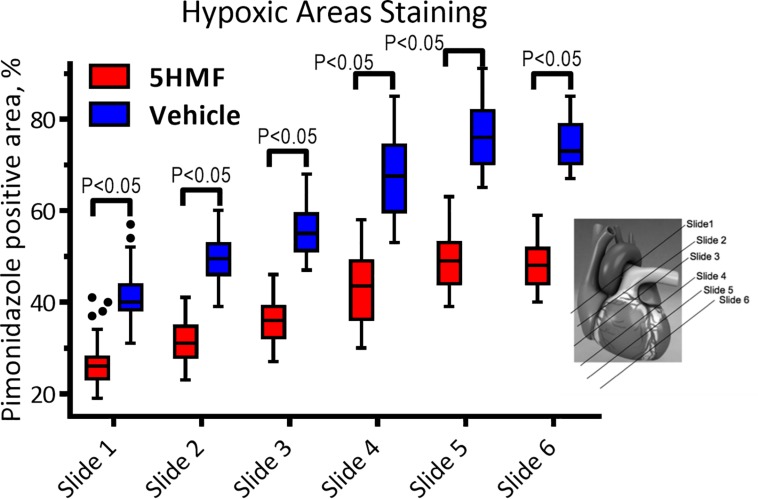
Heart staining for hypoxia with pimonidazole. Positive pimonidazole staining represents areas of hypoxia in the cardiac tissue. Amount of hypoxic areas increased with the proximity to the heart apex.

5-HMF-induced increased O_2_ affinity and reduced positive hypoxic staining by pimonidazole compared to the vehicle group (Figure 4). In addition, sections closer to the apex were drastically more hypoxic in the vehicle compared to 5-HMF. At the base of the heart, 41 ± 7% and 27 ± 5% of the tissue were hypoxic for the vehicle and 5-HMF group, respectively. The apex of the heart showed 74 ± 6% and 48 ± 5% positive staining for the vehicle and 5-HMF group, respectively. Animals treated with 5-HMF had statistically less hypoxic tissue per slide when compared to vehicle group (*P* < 0.05). On average, 5-HMF reduced hypoxic tissue by 60%.

### Oxygen Distribution During Acute Hypoxia

**FIGURE 5:**
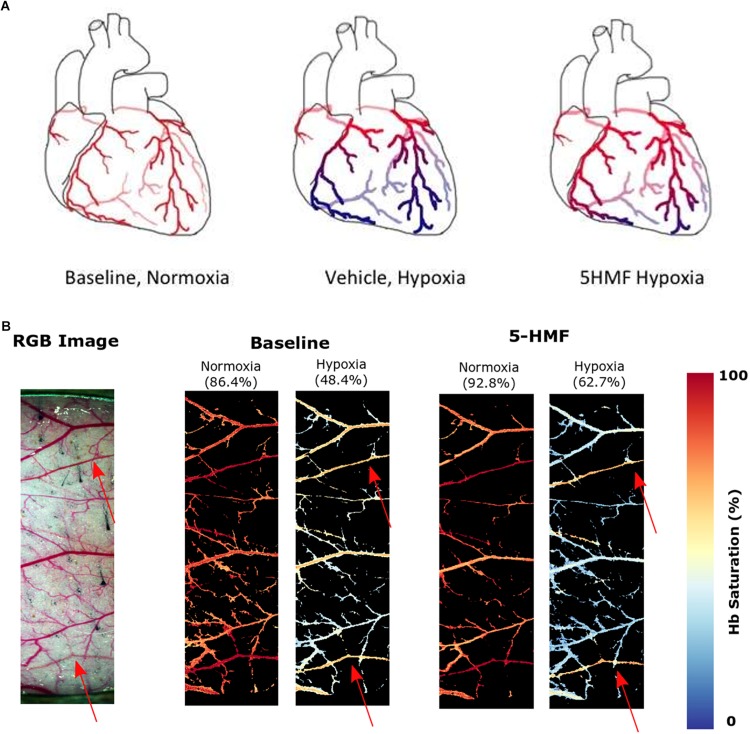
**(A)** Increasing Hb-O_2_ affinity during hypoxia with 5-HMF preserves cardiac oxygenation. Representation of the oxygenation in the coronary circulation during normoxia and hypoxia. Red represents oxygenated blood, blue represents deoxygenated blood. Left, during normoxia, cardiac tissue is well oxygenated. Center, during hypoxia, O_2_ is offloaded to the tissues. Right, Increasing Hb-O_2_ affinity during hypoxia with 5-HMF increases Hb-O_2_ saturation during hypoxia and increases coronary O_2_ delivery. **(B)** Dorsal skinfold window chamber HSIs. Left, RGB representation of the analyzed region of the chamber. Center, resulting hemoglobin (Hb) saturation maps at normoxia and hypoxia prior to the administration of 5-HMF. Right, resulting Hb saturation maps at normoxia and hypoxia after administration of 5-HMF. Colormap for the saturation is presented on the right. Arterial saturation, as measured by an arterial line blood sample, for each timepoint, is shown in parenthesis. Arterioles in each image are represented by red arrows.

The saturation maps from the hypoxia experiment are shown in Figure 5B. Consistently, arterial Hb saturation from blood gas analysis was 62.7% with 5-HMF and 48.4% with vehicle during hypoxia. Studying the Hb-O_2_ saturation in the dorsal window using HSI indicates that microvascular saturation during normoxia were distributed between 60 and 90% for venules and arterioles, and decreased to 30 to 50% during hypoxia. Treatment with 5-HMF during normoxia does not appear to affect Hb-O_2_ saturation of arterioles and venules in the dorsal window. However, during hypoxia 5-HMF shifted the Hb-O_2_ saturation distribution toward the arterioles, increasing arteriolar Hb saturations to 60%, and decreasing the venular Hb saturations to 20%.

### Heart Perfusion Studies

**FIGURE 6:**
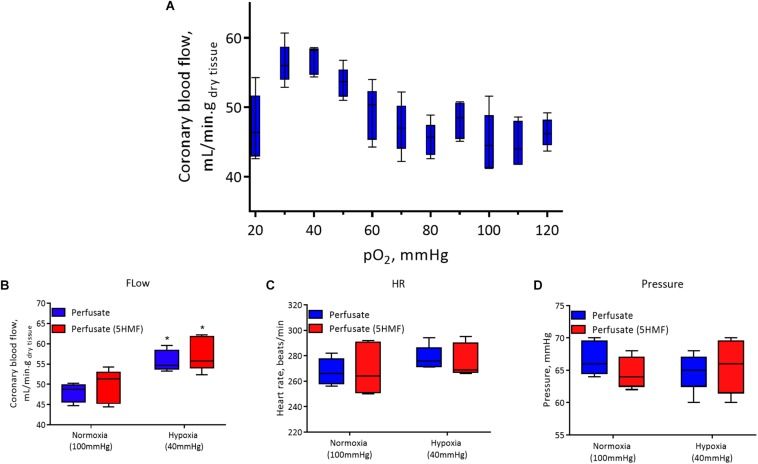
In all panels, control perfusate is shown in blue and 5-HMF containing perfusate is shown in red. **(A)** Coronary blood flow in the isolated murine heart at different oxygen tensions with control perfusate. **(B)** Coronary blood flow. **(C)** Heart rate (HR). **(D)** Left ventricular pressure at normoxia and hypoxia for control perfusate and 5-HMF containing perfusate. ^∗^ represent statistically significant differences (*P* < 0.05) relative to normoxia.

**FIGURE 7:**
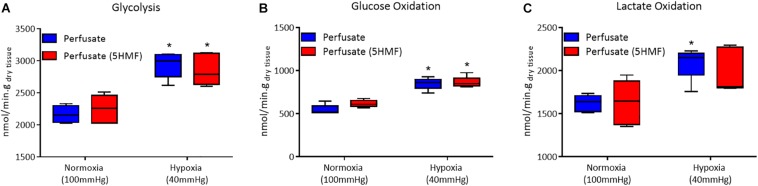
In all panels, control perfusate is shown in blue and 5-HMF containing perfusate is shown in red. **(A)** Glycolytic. **(B)** Glucose oxidation. **(C)** Lactate oxidation rates at normoxia and hypoxia for control perfusate and 5-HMF containing perfusate. ^∗^ represent statistically significant differences (*P* < 0.05) relative to normoxia.

Isolated rat hearts were studied with control perfusate and with perfusate containing 3mM 5-HMF. Coronary blood flow increases as the O_2_ tensions of the perfusate was decreased, with a max coronary blood observed around 30 to 40 mmHg (Figure 6A). Hypoxia (perfusate equilibrated at 40 mmHg) increased coronary blood flow increased compared to normoxia (perfusate equilibrated at 100 mmHg); although no differences were detected in HR or developed pressure during hypoxia and normoxia (Figures 6B,C). Addition of 3mM 5-HMF to the perfusate did not have any effect relative to the use of the perfusate alone, suggesting that 5-HMF does not have any pharmacological effect in the heart (Figures 6B,C). Glycolysis, glucose oxidation and lactate oxidation rates increased during hypoxia (perfusate equilibrated at 40 mmHg) compared to normoxia (perfusate equilibrated at 100 mmHg) (Figures 7A–C). Addition of 3mM 5-HMF to the perfusate did not have any effect on Glycolysis, glucose oxidation or lactate oxidation rates relative to the use of the perfusate alone (Figures 7A–C). Therefore, 5-HMF does not appear to have any cardiac metabolic effect.

## Discussion

The principal finding of this study was that 5-HMF decreased hypoxic damage experienced by the heart due to severe hypoxic hypoxia. Exposure to hypoxia had the expected effect on the vehicle group, with an increase in CO at first exposure to hypoxia (15% O_2_). This corresponds to a moderate level of hypoxia comparable to what is observed at altitudes of 2400 m, where the increase in cardiac activity is a common response to preserve tissue oxygenation (Wyss et al., 2003). As hypoxia severity increased to 10% O_2_ and later to 5% O_2_, the cardiac tissue started to fail. The mechanical ability of the heart to pump blood became impaired in the vehicle control group. This is most evident in the decline of CO, EF, SV, SW, and the considerable amount of cardiac hypoxic tissue areas seen in the vehicle group. On the other hand, treatment with 5-HMF resulted in mild changes in cardiac function at 15% O_2_ hypoxia. However, 5-HMF-induced increases in Hb-O_2_ affinity preserved, and in certain cases increased, cardiac indices of function, including CO, EF, SV, and SW at both 10% and 5% O_2_ hypoxia, compared to the vehicle group. However, the most notable changes in cardiac function were observed at 5% O_2_ hypoxia. In small rodents, the heart appears to compensate by increasing SV rather than HR in response to hypoxia. One explanation for the lack of chronotropic response in response to hypoxia is the cardio-depressive effect of the anesthesia isoflurane, since previous hypoxia studies in conscious animal showed increases in HR with hypoxia. Animals experienced a slight increase in MAP at 15% O_2_; however, as O_2_ levels decreased, MAP decreased as well. There are no significant differences between the treatment groups. Additionally, by 5% FiO_2_ both treatments converge to a MAP of 60 mmHg. Since there were no significant changes in MAP, but there were improvements in CO and SV at 5% in the presence of 5-HMF, there seems to be a volume dependent effect induced by the 5-HMF during hypoxia, whereas the pressure dependent effect appears to be determined by the SVR.

Vascular resistance significantly decreased for the 5-HMF group, but not for the vehicle group relative to normoxia. As multiple signaling pathways are activated by hypoxic stimuli (such as O_2_, CO_2_, and lactate), they are integrated in different vascular beds to modulate the response to hypoxia. Mild reduction in lactate levels from the 5-HMF induced increase Hb-O_2_ affinity during hypoxia appears to reduce the severe hypoxia-induced mixed blood acid-base disorder observed in the vehicle group. The hypoxic protective effects of 5-HMF-induced increase in Hb-O_2_ affinity were more evident at 5% O_2_ hypoxia, when O_2_ upload in lungs was drastically challenged.

Effective arterial elastance (Ea), which reflects the vascular impedance and is calculated as the mean end-ejection arterial pressure normalized by the ventricular end-systolic pressure (Kass and Kelly, 1992), correlated with the SVR changes. The partial blunting of the measured SVR response to hypoxia in the vehicle groups is part of the compensatory mechanisms to preserve vital organ O_2_ delivery by preferentially perfusing vital organs (Cabrales et al., 2008; Tomasco et al., 2010). The ratio of end-systolic pressure (Pes) to SV was constant under a given steady state vascular impedance load and is indicative of ventricular-arterial coupling. Additionally, during severe hypoxia, ventricular-arterial coupling for the 5-HMF group was significantly lower compared to normoxia and compared to the vehicle group during severe hypoxia. Although the SV increased for the 5-HMF group, the end systolic pressure is unresponsive to hypoxia. These results are consistent with previous findings that increasing Hb-O_2_ affinity during hypoxia resulted in a significant increase in the diameter of arterioles in the microcirculation (Dufu et al., 2017). This observed vasodilation is one explanation for the differences in SVR and Ea between groups, since the increased diameter can improve blood flow, and therefore, oxygen delivery.

Treatment with 5-HMF induced an increase in Hb-O_2_ affinity and a decrease in P50 relative to the vehicle group. Since the hypoxic challenge used in the study was controlled based on the inspired partial pressure of O_2_, blood O_2_ content rather than pO_2_ becomes the critical determinant of tissue O_2_ delivery to tissues (Yalcin and Cabrales, 2012). 5-HMF-increase in SaO_2_ translated into an improved O_2_ delivery to tissues, as suggested by Figure 3I. Histology analysis of the heart shows that 5-HMF was effective in protecting the myocardium from becoming hypoxic during hypoxia. However, positive pimonidazole staining increased toward the apex of the heart in both vehicle and 5-HMF group. This result was expected due to the morphology and hemodynamics of coronary circulation, in which the coronaries bring oxygenated blood from the aortic sinus to provide O_2_ to the myocardium. As such, regions farthest from the aortic sinus will encounter the most significant deoxygenation. The apex of the heart performs the most work, resulting in higher metabolic demands, and thus faster oxygen depletion. By increasing the oxygen affinity, 5-HMF increased O_2_ loading, offering better oxygenated blood to cardiac tissue, especially where metabolic demands are highest. A graphical representation of the expected oxygen distribution in the coronary circulation is shown in Figure 5A. The protective effect of increased O_2_ affinity is evident as the total average of positive staining in the vehicle group was nearly 60%, versus the approximate 40% positive staining for the 5-HMF group. By pharmacologically shifting the SaO_2_ curve to the left, 5-HMF reduced cardiac hypoxic tissue by nearly 60% during hypoxia.

The changes in microvascular dorsal skinfold saturation (Figure 5B) also support the hypothesis that increased O_2_ affinity allows for better oxygenated blood to arrive the more hypoxic regions. Prior to 5-HMF administration, the distribution of O_2_ during hypoxia appears relatively equal between arterioles and venules. On the other hand, the O_2_ distribution during hypoxia after 5-HMF infusion appears higher in the arterioles than venules. As arterioles are prior to venules in the circulatory system, the oxygen unloading has to be superior in the presence of 5-HMF in order for the venules to have a much smaller saturation during hypoxia as compared to without 5-HMF. Furthermore, since the arterial saturation, as measured through the arterial line, was larger in the presence of 5-HMF, the decreased venule saturation is not a result of a decreased amount of oxygen in circulation. Due to the allosteric binding/unbinding of O_2_ to Hb, if the Hb saturation is higher when the blood arrives to the less hypoxic regions, then the unloading will be less than if the saturation was lower. This secures most of the O_2_ to the Hb as blood flows through the less hypoxic regions. As blood reaches the more hypoxic regions, the oxygen gradient is now sufficient to allow unloading from Hb, at which point the allosteric unloading takes place, and the majority of the O_2_ is then delivered to the more hypoxic tissues. As blood arrives to the venous circulation, the Hb saturation is then very low. This low venous Hb saturation can lead to a nitric oxide dependent vasodilation of capacitance vessels (Redfors et al., 2014), resulting in the observed decrease in SVR. In contrast, in the absence of 5-HMF, Hb’s O_2_ affinity is lower, resulting in a larger O_2_ unloading in the less hypoxic regions. As blood reaches the more hypoxic regions, the gradient is still large, however, the allosteric unbinding effect will be smaller, resulting in less O_2_ unloading, resulting in an increased presence of O_2_ in the venous circulation as shown in Figure 5B. Despite these effects being studied in the skin, it is likely for them to generalize to the coronary microcirculation as shown in Figure 5A.

The isolated rat heart studies further support the idea that the protective effect of the 5-HMF is a result of the increased O_2_ affinity and not direct effects in coronary flow or cardiac muscle metabolism. The use of an isolated heart model eliminates confounding neurohumoral factors, as the resulting cardiac function will be entirely dependent on the contents of the perfusate. In the absence of blood, perfusate with 5-HMF does not have any effects on cardiac performance. The absence of significant differences in coronary flow, HR, and pressure between the control perfusate and 5-HMF containing perfusate (Figures 6B–D) suggests that there is no change in cardiac function in the absence of the effects of increased Hb-O_2_ affinity. The similarity in coronary blood flow in the presence and absence of 5-HMF in the perfusate is consistent with the claim that the observed decrease in SVR in the presence of 5-HMF is a result of vasodilation due to the increased Hb-O_2_ in the arterial circulation, and to vasodilation in the venous circulation due to decreased venous saturation, not due to a vasoactive effect of 5-HMF.

The isolated heart metabolic analysis is also supportive of the fact that there is no change in cardiac muscle metabolism in the presence of 5-HMF. The preserved glycolytic, glucose oxidation and lactate oxidation rates between control and 5-HMF containing perfusates, in either hypoxic or normoxic states, suggests that there is no direct effect of 5-HMF in cardiac muscle glycolysis or glucose metabolism. Glycolysis is the predominant source of energy during hypoxia (Whitmer et al., 1978). Therefore, 5-HMF having no direct effect over glycolysis is evidence that the superior cardiac function during hypoxia and in the presence of 5-HMF, as measured by CO, SW, SV, and EF, are a consequence of the decreased hypoxic damage in the presence of 5-HMF due to increases in Hb-O_2_ affinity in the myocardial circulation, and not direct effects of 5-HMF in myocardial metabolism.

In light of the results of this study, the possible health benefits mediated by 5-HMF (e.g., from the diet) should be considered. Information concerning the human daily dietary 5-HMF exposure is scarce. Daily 5-HMF consumption data is rare and previous research estimated a human ingest up to 150 mg of 5-HMFper day (Ulbricht et al., 1984); however, in another recent report, it was suggested that a mean 5-HMF intake of 6 mg per day was estimated for Spanish adolescents (Delgado-Andrade et al., 2007). Intake of Honey, Jam, Fruit cake, Tomato paste, Ketchup, Syrup, Fruit juice, Canned fruit, and other foods containing 5-HMF in sufficient amounts could increase Hb-O_2_ affinity and preserve O_2_ transport to cardiac tissue during hypoxia. Although to achieve therapeutic doses of 5-HMF from foods, the amount of food required would need to be significant.

One potential limitation of this study is that the experimental results with fossorial animals, such as hamsters, could not be extrapolated to other mammals and humans. While their arterial pO_2_ at normoxia is lower compared to other rodents and humans, their responses to hypoxia and hypercapnia are more similar to those of non-fossorial rodents (Schlenker and Goldman, 1985; Tomasco et al., 2010). At low O_2_ concentrations, their respiratory system responds by increasing the frequency of respiration, with no changes in tidal volume and little activation of stretch receptors (Tomasco et al., 2010). In the experimental setup used in this study, animals were connected to a mechanical ventilator, running at a constant frequency with slight increases in peak inspiratory pressures as O_2_ concentrations decreased. Therefore, specific ventilatory responses to hypoxia in hamsters were dampened by the used of mechanical ventilation. Hypoxia-induced reduced metabolism has been shown to occur only in small species, as allometric variation (mass-specific metabolic rate and O_2_ composition to body mass) is higher in smaller species as compared to larger-size species (Kleiber, 1961). Therefore, direct extrapolation of the degree of protection to hypoxia that increases in Hb-O_2_ affinity provide still needs to be evaluated in larger animals and humans. Another limitation includes the cardioprotective effects of isoflurane, which has been shown to help preserve cardiac function under different conditions (Yildirim et al., 2009; Redfors et al., 2014). The improvement in cardiac function in the presence of 5-HMF compared to the vehicle control group indicates that there is a beneficial improvement in cardiac function due to 5-HMF in addition to any isoflurane cardioprotection effect, as any isoflurane cardioprotection during hypoxia is present in the vehicle group. Awake animal studies are required to determine the significant benefits of 5-HMF in cardiac function independent of isoflurane. The measurement of Hb-O_2_ affinity of blood samples require dilution in a strongly buffered solution (TCS Hemox solution, pH 7.3) and deoxygenation in the absence of CO_2_. Therefore, the measurements of Hb-O2 affinity and p50 values measured with the TCS Hemox Analyzer do not reflect the Bohr and Haldane Effects, which are expected to further decrease the Hb-O_2_ affinity as a function of the acid-base status *in vivo*. Previous studies have replicated pH and pCO_2_ changes induced by 10 and 5% O_2_ hypoxia in order to better replicate the *in vivo* conditions eliminated by the TCS Hemox Analyzer buffer and principle of operation. Results indicated that the P50 values, independent of blood pCO_2_ and pH, are lowered by 3% at 10% O_2_ hypoxia and by 4% at 5% O_2_ hypoxia (Zwart et al., 1982). Study of LVCF in small animals are limited by their high HR which shortens endocardial perfusion (Collins et al., 2003). Another important note is that because their baseline HR is already high, there is a limited rise in CO when needed. The CO response can potentially be different in larger mammals, as there is more flexibility in HR.

## Conclusion

This study exposes that relatively small amount of 5-HMF preserved LV cardiac function and reduced myocardial hypoxia during severe hypoxic hypoxia (5% O_2_). Moreover, 5-HMF-induced increase in Hb-O_2_ affinity improved tolerance to 5% O_2_ hypoxia exposure. Therefore, 5-HMF-induced increase in Hb-O_2_ affinity increased O_2_ delivery during hypoxia, thus preserving MAP, HR, LVCF, and reduced lactate and myocardial hypoxia. 5-HMF-induced increase in Hb-O_2_ affinity increased blood O_2_ loading in the lungs and allowed for increased O_2_ delivery during hypoxia. The increase in O_2_ delivery during hypoxia also decreased hypoxia in the myocardium. This can be observed in the decreased hypoxic staining of the myocardium in the presence of 5-HMF. The isolated heart experiments suggest that 5-HMF is not conducive to changes in myocardial glucose metabolism, suggesting that the decreased hypoxic damage is a consequence of the increased affinity. Furthermore, the hyperspectral analysis of the microcirculation suggests increased oxygen delivery in the presence of 5-HMF, further supporting the hypothesis that the decreased hypoxic damage is tied to the 5-HMF-induced increase in oxygen affinity of hemoglobin.

## Data Availability statement

All datasets generated for this study are included in the article/supplementary material.

## Ethics Statement

This experimental protocol was approved by the UCSD Institutional Animal Care and Use Committee. This research was conducted in compliance with the US Animal Welfare Act and other federal statutes and regulations relating to animals and experiments involving animals and adheres to principles stated in the Guide for the Care and Use of Laboratory Animals, NRC Publication, 2011 edition.

## Author Contributions

EA and PC designed the experiments, and acquired and analyzed the data. All authors participated in the data analysis and drafting of the manuscript, and read and approved the final manuscript.

## Conflict of Interest

The authors declare that the research was conducted in the absence of any commercial or financial relationships that could be construed as a potential conflict of interest.
